# The Macroeconomic Imbalance Procedure at the Heart of EU Economic Governance Reform

**DOI:** 10.1007/s10272-022-1028-7

**Published:** 2022-02-12

**Authors:** Willi Koll, Andrew Watt

**Affiliations:** 1 Bonn, Germany; 2 Department of the Hans-Böckler Foundation, Macroeconomic Policy Institute (IMK), Hans-Böckler-Straße 39, 40476 Düsseldorf, Germany

**Keywords:** E61, E62

## Abstract

While the euro officially came into being in 1999, it was the introduction of euro notes and coins 20 years ago in January 2002 that made the common currency a tangible reality for European citizens. The circle of member states has since grown from 11 to 19, and a growing section of the population no longer has any personal experience with a “national” currency, yet the debate on the legal and institutional framework underpinning the common currency has never gone away.

Substantial reforms were introduced in the wake of the near-death experience of the euro crisis. A calmer period after 2012's "whatever it takes" came to an end, however, as the coronavirus crisis ravaged national economies and public budgets. The European Commission had launched an economic governance review process at the start of 2020, but this was swept aside by the pandemic. The fiscal rules were suspended. On the other hand, a number of important new initiatives were taken, e.g. the Green Deal, the European Instrument for Temporary Support to Mitigate Unemployment Risks in an Emergency (SURE) programme, the pandemic emergency purchase programme (PEPP), and the Recovery and Resilience Facility (Watt, 2020; Watzka and Watt, 2020). Meanwhile, "the correction of macroeconomic imbalances has been interrupted and new vulnerabilities are emerging, highlighting the importance of preventing and addressing risks and divergences in a timely way" (European Commission, 2021, 9).

In the autumn of 2021, the Commission relaunched the process, inviting stakeholders and citizens to make submissions to a consultation process by the end of 2021. The suspension of the rules is due to be terminated at the end of 2022, and it is widely recognised that the existing rules cannot be reapplied, not least in view of the substantial (around 15 percentage point) rise in public debt and the heightened public investment needed to achieve decarbonisation. The Recovery and Resilience Facility is politically important, but on its own clearly inadequate for this task. The French Presidency has scheduled a summit for March at which, hopefully, a top-level political agreement on reforms can be reached.

**Willi Koll**, Independent researcher and writer, Bonn, Germany.

There is no shortage of proposals to reform the fiscal rules (Dullien et al., 2021). In this article we focus on an under-discussed, but, we argue, crucial aspect of the economic governance framework and its necessary reform: the "macroeconomic imbalances" between member states. Lacking the adjustment mechanism of a variable exchange rate, it is vital to ensure that unwarranted price and wage inflation differentials between member states – and consequent negative competitiveness and demand spillovers between them, resulting in harmful current account imbalances and macro-financial risks – are avoided. Much more so than sticking to (arbitrary) targets for government deficits and debts.

## The Macroeconomic Imbalance Procedure

In its economic governance review Communication of February 2020, which launched the economic governance reform process, the European Commission (2020, 5) noted that the main objectives of the policy regime, in addition to "ensuring sustainable government finances" include "avoiding macroeconomic imbalances,…closer coordination of economic policies in particular in the euro area, and…promoting the convergence of economic performances among Member States".

The Macroeconomic Imbalance Procedure (MIP) was set up in 2011, as part of the "six-pack" of reforms after the first wave of the euro crisis.[1] It marked recognition that the origi-

1 The MIP consists of two EU Regulations: No 1176/2011 on the prevention and correction of macroeconomic imbalances and No 1174/2011 on enforcement measures to correct excessive macroeconomic imbalances. For an overview, see European Commission (2016).

nal Maastricht approach, which relied on market pressures to correct incipient imbalances, via a change in relative competitiveness, had failed. This so-called real exchange rate channel had for years been overruled by a pro-cyclical real interest rate channel, a self-sustaining dynamic interplay between fast and slow-growth member states and their often pro-cyclical fiscal stances. Dynamically growing economies experienced higher nominal wage and price inflation, which meant low real interest rates, fuelling growth further. Fast nominal demand growth meant that fiscal rules were not binding. In slow-growth economies, it was the opposite: high real interest rates and austerity pressure from the fiscal rules. The associated permanent build-up of external imbalances – current account deficits in the former, surpluses in the latter – and the consequent debtor-creditor relations between countries were unsustainable. After the “sudden stop” crisis, imbalances were “corrected” asymmetrically via hugely costly and one-sided deflationary austerity policies. The asymmetric rebalancing led, among other things, to the euro area as a whole running a large current account surplus vis-à-vis the rest of the world.

Taking its cue from the Stability and Growth Pact (SGP), under the MIP a member state with imbalances is subject to surveillance under a preventive arm and, eventually, the Excessive Imbalance Procedure (EIP), as a corrective arm, which in the case of euro area members can lead to sanctions. In practice, however, sanctions have not been imposed. Unlike the SGP, there are no clear triggers for the various steps in the legal process. Countries are initially assessed against a scoreboard of quantitative indicators in an Alert Mechanism Report (AMR). This is not a mechanical process, however; it acts as a filter to select countries for so-called in-depth reviews. These reviews form part of the European Semester process, which issues recommendations under the MIP as part of the country-specific process.

MIP surveillance has contributed to a supportive policy environment in a number of respects, e.g. by raising awareness about the issues around macroeconomic imbalances, providing a framework for prioritising the national policy agenda with a view to ensuring macroeconomic stability, and strengthening policy dialogues on macroeconomic stability issues between national authorities and EU institutions as well as among member states (European Commission, 2020, 43). However, the MIP suffers from a number of serious weaknesses.

The fundamental architecture of the MIP is asymmetric. (e.g. European Commission, 2020, 13; Bénassy-Quéré and Wolff, 2020, 11; Dullien et al., 2020, 17). The selection of indicators in the scoreboard and the threshold values chosen – e.g. a -4% of GDP floor for current account deficits but a +6% ceiling for surpluses and the fact that there is only an upper limit on nominal unit labour cost increases – places adjustment pressure one-sidedly on deficit countries with above-average inflation. At the aggregate level this manifests itself as a tendency towards an overall euro area current account surplus and persistent low inflation, which makes the relative adjustment of competitive positions highly costly (Bénassy-Quéré and Wolf 2020, 13; Koll and Watt 2018, 18).

The MIP follows a country-by-country approach that pays too little attention to defining an overall macroeconomic policy stance for the euro area, and then clearly identifying the role to be played by different member states within that overall frame (European Commission, 2020, 18; European Parliament, 2021, §50; Bénassy-Quéré and Wolff 2020, 11).

The MIP confuses “competitiveness”, in a general sense, with the specific problem of intra-EMU (or at least intra-EU) imbalances. In the scoreboard, the real exchange rate is measured against a basket of trading partner currencies and is thus affected by movements in the external value of the euro over which countries have essentially no control.

The complexity of the indicators in the scoreboard is problematic and their internal coherence and relevance questionable (European Parliament, 2021, §50; Bénassy-Quéré and Wolff 2020, 18-19; Dullien et al., 2020, 18). The scoreboard in its latest intage has 14 main quantitative indicators with thresholds, supplemented by no less than 28 auxiliary indicators (European Commission 2020b, 7). The sheer number of main indicators, on paper of equal worth, makes it hard to clearly distinguish situations of genuine concern. A number of indicators are irrelevant to the issue of macroeconomic imbalances within the euro area (or EU). The inclusion of specific employment indicators, in addition to the overall unemployment rate, has further muddied the waters. Among the internal imbalance indicators, house prices are a sectorally specific indicator, while the government debt ratio is part of the fiscal rules. On the other hand, what is arguably the most important yardstick – the divergence over time of national inflation from the price stability target of monetary policy and thus, together with divergence in the real interest rate, the cumulative shift in the real exchange rate within the euro area – is not incorporated.[2]

Partly reflecting the plethora of indicators, there is a lack of transparency about the surveillance process that follows the evaluation in the scoreboard: the in-depth reviews of selected countries. There are no clear criteria for distinguishing between imbalances and excessive imbalances, and the European Commission itself (2020, 14) sees a need to improve the

2 The indicator referring to the real effective exchange rate is against world trading partners and reflects the change over the last three years only, while nominal unit labour costs – which within a monetary union do represent an indicator of the real exchange rate – are measured absolutely and not in relation to the average or a suitable benchmark.

link between the MIP analysis and the policy recommendations it gives to member states. The surveillance procedure becomes a bargaining game between the dedicated units in the European Commission and specialised parts of national bureaucracies, without public visibility or participation. In short, there is a lack of "ownership".

The Excessive Imbalance Procedure (EIP), which would require the delivery of a corrective action plan to be carried out within a pre-determined timeframe and would certainly lead to a political debate in the member state concerned, has never before been activated. The EIP is only the first step needed before sanctions can be imposed on euro area countries or repeated lack of compliance. It is all too apparent that the MIP process has shied away from imposing constraining measures on member states, despite substantial imbalances (Bricongne and Turrini, 2017).

All of the above factors contribute to a general assessment that the MIP lacks the "political traction necessary" (European Commission, 2020, 13) and an adequate degree of effectiveness in steering member state behaviour (Bénassy-Quéré and Wolff 2020, 14, 17).

Last but not least the relationship between the MIP and the fiscal rules is unclear and potentially inconsistent (Koll and Watt 2018, 12; European Fiscal Board, 2019, 91). The process of fiscal monitoring and that of monitoring imbalances are separate. Yet policy recommendations for the one will certainly affect outcomes for the other. And it is conceivable that recommendations under the two processes will be contradictory. The fact that the EIP has never been activated as well as the more specific Treaty injunction to "avoid excessive deficits" indicates an imbalance between the two processes in favour of the fiscal rules, although this is not justified from a purely economic point of view.

## Strengthening and reforming the MIP process

In the light of the above, an effective process that sought to coordinate member state economic policies with a view to avoiding and correcting imbalances would need to meet a number of requirements. In particular, it should have a clear and relevant benchmark, be symmetric in approach, identify in a forward-looking way imbalances that pose a risk to other countries (cross-border spillovers), be consistent with the fiscal rules and be effective in influencing member state behaviour where required, focusing on "gross errors", while avoiding unnecessary interference in national policy choices.

While it is not possible to move swiftly from the current situation to an optimal policy regime, a package of reforms that would generate substantially improved outcomes is achievable, and the current reform discussion is an important opportunity to make necessary changes. It is useful before detailing specific reforms to restate some fundamental issues (Koll and Watt 2019, 14-20).

The critical macroeconomic stability condition (given initial equilibrium) is for domestic prices and nominal unit labour costs to move, in the medium run, in step with the price stability target of the central bank in each member state. This avoids changes in real exchange rates and equalises short-term real interest rates.[3] Both avoid divergences in both internal demand and price competitiveness, and thus work through "price" and "quantity" channels to push current account positions (within the monetary union at least) towards balanced positions. Boom-bust cycles and the build-up of "toxic" debtor-creditor relations between member states are avoided. It also maintains the functional distribution of income at the national level with workers reaping productivity gains in their real wages. Given that this also implies that the price stability target of the central bank is achieved in aggregate, monetary policy can – and according to the Treaty must – set policy to support sustainable growth and employment. This policy, in turn, creates a favourable environment for public finances, which reduces the risk of "excessive" deficits and debts on the part of fiscal policy as well as the likelihood of having to impose corrective fiscal rules. Sustained deviations from these benchmarks, unless necessary to correct a prior imbalance, have cross-border impacts (either direct or via monetary policy) that can be serious. Policy coordination of a constraining kind is therefore justified. It is much more important to constrain member state policies here than is the case with the often detailed policy recommendations currently issued to countries under the European Semester.

Member states have the tools at their disposal to meet the benchmarks. They can use fiscal policy, with the important caveat that fiscal rules do not force them to do otherwise. Despite a centralised monetary policy, they can use macroprudential policies to influence demand trajectories in specific financial and asset markets (most notably housing). Furthermore, to an extent depending critically on national institutional structures, they can influence the trajectory of nominal prices and, particularly, wages.[4]

3 Strictly speaking: on safe assets. However, by meeting the condition for tension-free growth, interest rate risk premiums and thus also differentials in real market rates would also be kept within narrow bounds.

4 While a debate has recently emerged about the use of direct price controls (following Weber, 2021), and anti-trust and other "price policies" may have a role to play, the more important element is generally collective bargaining instruments and government measures to influence nominal wages. These include (inter)sectoral agreements, minimum wages, benchmarking against other countries, *erga omnes* rules, government procurement rules. For a discussion and literature references, see Koll and Watt (2019, 2021).

These three sets of instruments are not independent of one another and require coordination at the national level. To this end, the relevant actors – national fiscal authorities, i.e. governments, national central banks,[5] and representative bodies of employers and labour unions – need to be brought around the table to discuss consistent and efficient strategies while retaining autonomy and independence. The balance between the measures deployed will vary from country to country depending on its institutional preconditions (not least, but not only with regard to its wage setting modalities). This is rightly a matter of national sovereignty. Monitoring by the EU should focus on critical *outcomes*, particularly cumulative price and unit labour cost trends and the current account.

## A feasible reform package

A series of measures could be feasibly taken to move in the direction of an economic governance mechanism that meets these requirements. While requiring some institutional creativity, the steps are legally undemanding, certainly compared to some of the reform proposals for the fiscal rules.

The obvious starting point and "lowest hanging fruit" is reform of the AMR scoreboard (Bénassy-Quéré and Wolff, 2020). All indicators used should be symmetrical in their implementation, and the number of indicators should be very substantially reduced and/or prioritised focusing on those which can be influenced by the macroeconomic policy actors. The decisive indicators are price and wage trends and the current account. Concretely we propose focusing the scoreboard on:

- the annual (expected) change in nominal unit labour costs (NULCs), with a symmetrical corridor (e.g. +/- 1%) around the ECB target of 2%
- the annual (expected) change in the GDP deflator, with the same symmetrical corridor
- the current account position, with a symmetrical corridor (such as +/- 3%) around a benchmark of zero percent of GDP
- the cumulative divergence in the price indicator from a suitable "equilibrium" starting point.

All other indicators would either be excluded or downgraded to supplementary contextual indicators. These choices are determined by a mix of conceptual but also pragmatic considerations.

In terms of the underlying economic logic, it is the intra-EMU current account that is relevant (for euro area members). This is not readily available, however. (An intra-euro area current account reporting should be examined). In the short run, the overall euro area current account position can be taken into consideration when evaluating that of each member state. In the medium run, given the aim of maintaining aggregate demand close to potential and floating exchange rates with most extra-EMU trading partners, the overall current account position of the currency area should itself not be substantially in surplus or deficit for extended periods.

The choice of the GDP deflator is because this is the equivalent on the price side to NULCs on the wage side and thus relevant in identifying income distribution developments. Because it is benchmarked around the ECB target and a balanced current account, an advantage of this approach, beyond its simplicity, is that it indicates the desired overall policy stance for the euro area. While the annual rate of change of the GDP deflator and NULCs provides a timely indicator of the direction of price competitiveness (and real interest rates) and has something of an advance warning characteristic, it essentially starts the clock anew every year. It is not sensitive to past accumulations of excessive or inadequate price development and can give misleading signals.[6] Hence, the use of the cumulative price change compared to that implied by the ECB target is recommended, serving as a simple real exchange rate indicator. In principle, methods could be deployed to determine the gap between the actual exchange rate and some measure of the equilibrium value (see the discussion in Bénassy-Quéré and Wolff, 2020, 19; and Couharde et al., 2017). However, such methods require, among other things, estimates of the output gap – which are well known from the debate on structurally adjusted fiscal deficits to be highly problematic as a policy guide. A pragmatic solution for euro area countries is to use index values for the price level starting from when the country joined the euro, benchmarked against a similar index path for the ECB target inflation rate. In 1999, current accounts for bigger member states were very small by subsequent standards, so this may be considered reasonable although for some (smaller) countries a different starting point may be appropriate.

The scoreboard would remain, as currently, a filter for the in-depth review, not a mechanical device generating policy recommendations. But the much greater focus and symmetry, with sensible benchmarks and a more forward-looking

5 As representatives of the European System of Central Banks and to the extent that they are responsible for national macroprudential policy. Where specialised agencies are involved, they could also be considered relevant actors.

6 After the euro crisis broke out, annual rates in countries such as Greece and Spain were substantially below the corridor, for example. Considered by itself, this would suggest a need for a more expansionary policy stance. But this would ignore the need to correct previous years in which prices and nominal wages increased in excess of the benchmark.

approach, would make the AMR a much more powerful and also visible tool, increasing transparency and ownership.

The policy recommendations to countries should be less detailed; but when they are made, they should have a more constraining effect. The MIP recommendations should be considered separately from those in the European Semester, which tend to include many supply side reforms of dubious cross-border relevance. The MIP recommendations to member states should be such as to be consistent with the overall stance required for the euro area as a whole. This should be set out in the section of the Country Specific Recommendations (CSRs) dealing with the euro area (see Bénassy-Quéré and Wolff, 2020, 33). The recommendations should primarily identify the contribution in terms of outcomes expected from each member state. Following the principle underlying EU directives, member states should be permitted as much leeway as possible as to how they achieve given objectives. They are best suited to do so and EU intervention should only be as constraining as necessary (subsidiarity). This is key to enhance ownership and compliance.

Given such a stripped down and symmetrical approach, there should be much less reluctance to invoke the so far unused EIP. Under the EIP, member states themselves have to draw up an action plan with a timetable for specific interventions to correct specific imbalances. In this way, policy discretion is left to the member states, but constraints or incentives can be imposed where they are needed to reign in negative or promote positive spillover effects.

Further steps are needed, however, to increase "ownership" and, specifically, to bring relevant actors around the table. As discussed above, these are national governments, national central banks (or other supervisory bodies) and the social partners. A coordinating body already exists – the Macroeconomic Dialogue of the EU (EUMED) – which brings these actors together (Koll, 2005). But it is weakly institutionalised, meeting twice a year at a technical and at a political level in Brussels. It lacks a "grounding" in national policies and a focus on the specific needs of the euro area. Greater traction of the MIP and more effective policymaking could be achieved by notably strengthening deliberative capacities, taking the MED as a starting point.

In particular, we propose establishing a dialogue specifically tailored to the euro area (EUROMED), with the participation of the finance ministers of *all* euro area member states, the EU Commission, ECB, and European representatives of social partners. Most importantly, each member state should set up a "MEDNAT" with the same representation of the corresponding national actors, as recommended also by the EP (European Parliament (2021), § 62). At all levels, MEDs should:

- discuss the overall national economic situation and prospects

- identify the need for economic policy action to achieve and maintain a macroeconomic policy mix of monetary, fiscal and wage policies that are oriented towards sustainable, tension-free growth and stability, in accordance with MIP requirements and country-specific recommendations

- identify the individual contributions of each actor and of their expectations regarding the respective contributions of the other macroeconomic actors in achieving this objective.

The EUROMED body would additionally have a coordinating function, notably to guard against any temptation to pursue beggar-thy-neighbour policies on the part of individual member states. Building on already existing bodies, the same functions might be provided by an extended informal Euro Group with social partners twice a year or by enlarging the existing EUMED with the presence of *all* finance ministers. As in the existing EUMED, in both fora a high level of consensus and trust among all key stakeholders is to be built up, whereas independence and autonomy of each policy actor is to be safeguarded. In the end, policy actors decide in their specific forum, but in light of the deliberations in the respective MEDs.

Support in the form of technical expertise could be provided to the deliberative institutions by adapting the mandate of the existing national productivity boards to focus more on "demand externality" and policy mix issues, rather than sticking to a supply side agenda (Koll and Watt, 2019, 25). Similarly, the European Fiscal Board could be given a wider remit.[7]

## Relationship between reform of fiscal rules and of MIP

This brings us, finally, to the issue of the appropriate relationship between the MIP – reformed and strengthened along lines proposed here – and the fiscal rules. Situations where a conflict can occur can be easily envisaged. In particular, a country can be constrained by debt or deficit rules to run contractionary fiscal policies, although expansionary policies are indicated by the MIP, e.g. on the basis of below-target inflation or a current account surplus. A reformed, more readily applied and effective MIP would contribute to the reduction over time of debt-to-GDP ratios and reduce the risk of corrective crises by encouraging, in a symmetric way, balanced economic growth at inflation

7 "Strengthening the existing EU Macroeconomic Dialogue (MED) and ensuring the link to (reformed) productivity boards, ..., would also enhance the effectiveness of the MIP" (Dullien et al., 2021, 28).

rates close to the ECB target. Thus the likelihood of countries coming up against the constraints of the fiscal rules is reduced. By constraining countries in "good times", a reformed MIP would complement the (existing) fiscal rules and help achieve what they, as is widely recognised, have not managed to do.

Reforms to the fiscal rules themselves are not discussed here (see Dullien et al., 2021 for a recent discussion), but a sensibly designed expenditure rule, for example, would make fiscal policy more symmetrically counter-cyclical, at least on the spending side. Conflicts between the MIP and reformed fiscal rules, at least in terms of the current fiscal stance, would thus be less likely. It is true, though, that this may not be the case considering the legacy of past debt. Countries are obliged to adjust towards a medium-term objective, which may conflict with cyclical stabilisation concerns. Fiscal rule reforms are likely to reduce, but not remove, this adjustment pressure. And it remains to be seen whether the necessary majorities for substantial reform of the fiscal rules can be mustered.

How might a potential conflict be defused and synergies between the processes promoted? The European Fiscal Board (2019) has tabled a proposal to make an explicit link between the fiscal rules and the MIP. Specifically, it proposes to do so

> by regulating the speed of adjustment towards the MTO in relation to Member States' macroeconomic imbalances. For example, Member States with large private debts and current account deficits could be required to speed up their adjustment towards the MTO or even to achieve a higher MTO. Conversely, Member States with persistent current account surpluses could be allowed to slow down their adjustment towards the MTO or to aim for a lower MTO. (European Fiscal Board, 2019, 55)

The reference here to the medium-term objective could be adapted to the case of an expenditure rule. The gap between the benchmark trajectory (e.g. real trend growth plus the ECB inflation target) and the concrete expenditure path of each country could be adjusted, in light of any MIP recommendation. For those countries for which macroeconomic imbalances have been identified, this would amount to a partial and time-constrained override of the fiscal rules. This would follow the same logic as the suspension of the fiscal rules during the pandemic, but for individual countries. The Council would ascertain that an exceptional circumstance – here the existence of substantial and persistent macroeconomic imbalances – pertains in a given set of countries. These would then be permitted to deviate from an adjustment path otherwise prescribed by the fiscal rules and follow instead a trajectory agreed upon by the Council.

This overall approach has been endorsed politically by the European Parliament (2021, §51-§52). It would stop well short of subsuming the fiscal rules under an economic policy coordination centred around the MIP. But it would amount to a temporary override of the fiscal rules for specific countries, which would require an agreement on a case-by-case basis by the Council. The principle could and should be operationalised in such a way that the extent of the "override" varies with the seriousness of the imbalance problem facing the country. Such an approach would go some way – depending on the precise modalities perhaps a considerable way – to anchoring a reformed MIP, and thus macroeconomically sensible policy orientations, within the economic governance framework, reducing the likelihood of fiscal rules producing sub-optimal and at times highly costly, destabilising outcomes.

## Conclusion

Balanced macroeconomic development is not only an essential goal by itself; it is also a prerequisite for achieving other important economic policy goals. For example, the phasing-out of fossil-based production and products as well as the move towards climate neutrality will subject the economies of all member states to a massive structural change. This change supposes strong acceptance by civil society. Therefore, the maintenance of a high level of employment and adequate incomes during this process is an indispensable condition. This prerequisite is doomed to fail if new dangerous macroeconomic imbalances develop between the member states again in the wake of the pandemic and a reinforced climate policy, leading to what Olivier Blanchard (2006) referred to as "rotating slumps" and painful corrective measures. A reformed MIP process in the sense described above can and must counteract this in a preventive way. Therefore, the Macroeconomic Imbalances Procedure should be at the heart of economic governance reform.

As for the reform of fiscal rules, there are very close links between macroeconomic balances and fiscal outcomes. Thus, a reformed MIP can act as a useful complement to a reformed Stability and Growth Pact and vice versa: balanced and sustained economic growth helps reduce government deficit and debt ratios while providing fiscal means for massive green public investment. By the same token, more effective counter-cyclical fiscal policies can contribute to a better macroeconomic stabilisation and balance in both good and bad times. The current French EU Presidency Programme's stated intention to give equal weight to both issues is most welcome: "It will take forward discussions on *the review of the EU's economic governance framework*, including analysis of the repercussions of the COVID-19 crisis on the European economy and on challenges it faces, *fiscal rules* and the *macroeconomic imbalances procedure*" (French Presidency of the Council of the European Union, 2022, 26). Hopefully this will be true in practice as well. Much of the MIP reform described above is legally undemanding and likely less a bone of political tension between member states than the fiscal issue. To this extent, MIP reform could be implemented ahead of changes to fiscal rules and could serve as a useful precursor of reforms.
